# Supplementary material for: Th17 cells contribute to combination MEK inhibitor and anti-PD-L1 therapy resistance in KRAS/p53 mutant lung cancers
Source: Nat Commun. 2021 May 10;12:2606. doi: 10.1038/s41467-021-22875-w (PMC8110980; doi:10.1038/s41467-021-22875-w)
Supplement: Supplementary file 1 — Supplementary Information [file 41467_2021_22875_MOESM1_ESM.pdf]

Th17 cells contribute to combination MEK inhibitor and anti-PD-L1 therapy resistance in KRAS/p53 mutant lung cancers

Peng DH and Rodriguez BL et. al

Supplementary Figure 1

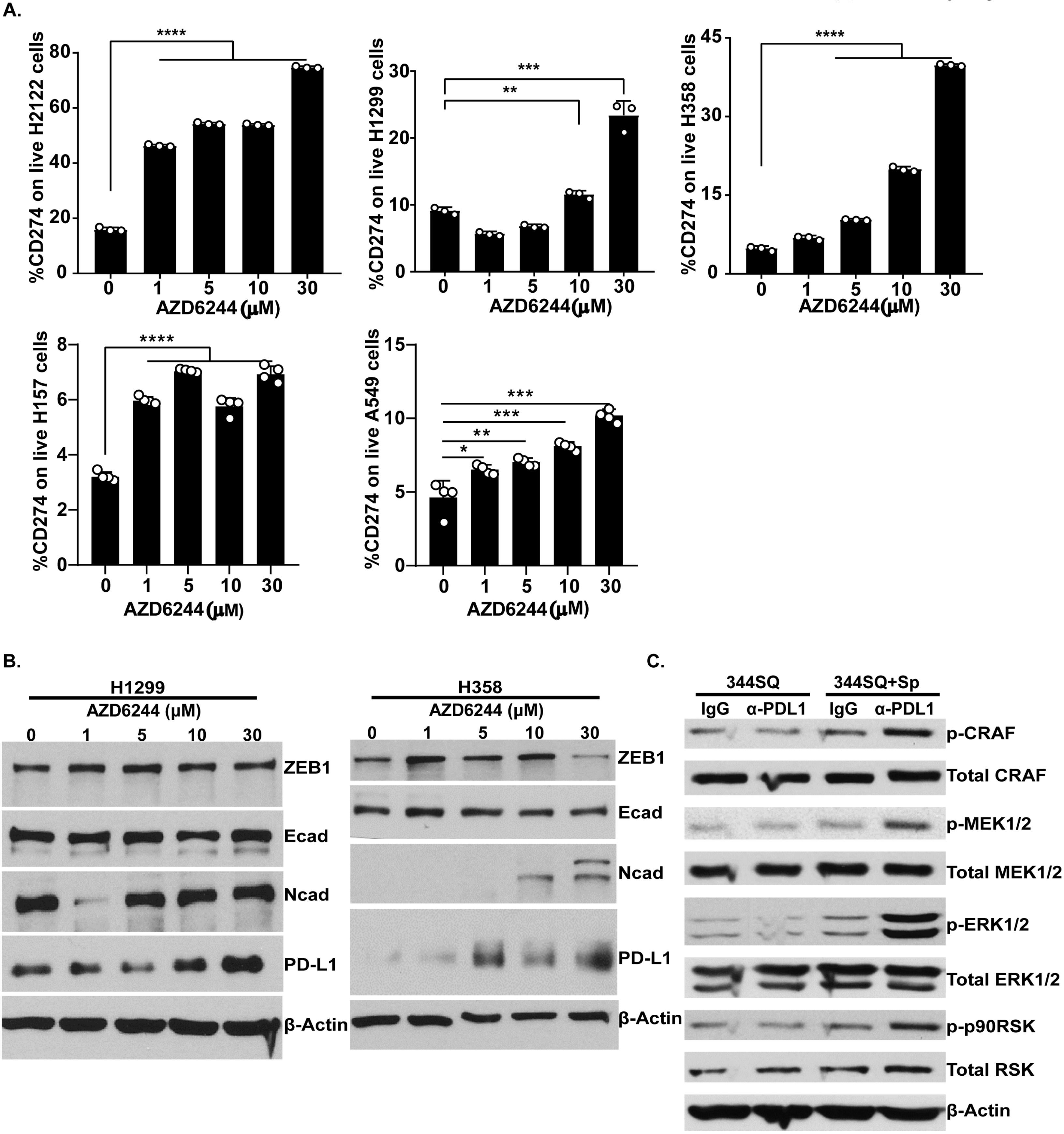

Supplementary Figure 1.

(A) Percent CD274+ (PD-L1) human lung cancer cell lines following 48 hr treatment with indicated concentrations of AZD6244 gated on live cells. Data are presented as mean values +/-SD. n = 3-4. Data were analyzed using unpaired Students *t test*. *, $P < 0.05$; **, $P < 0.01$; ***, $P < 0.001$; ****, $P < 0.0001$.

(B) Western blot of indicated proteins in H1299 and H358 human lung cancer cell lines following 48 hr treatment with indicated concentrations of AZD6244.

(C) Western blot of indicated proteins in 344SQ murine KP cell line cultured alone or in co-culture with splenocytes, treated with 20 μg/ml anti-PD-L1 blocking antibody for 96 hrs.

Supplementary Figure 2

Flow Cytometry sample gating strategy for total T-cell populations

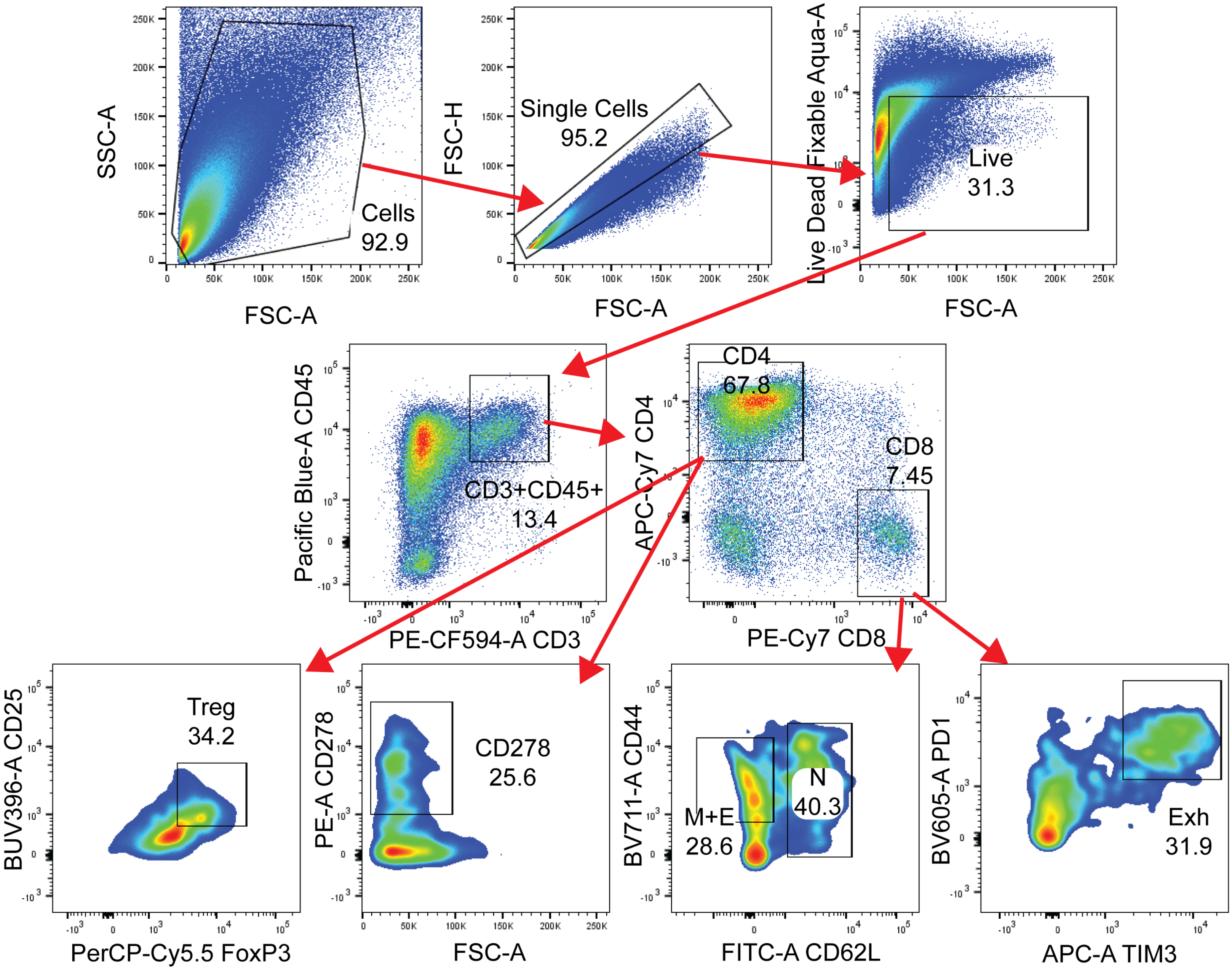

Supplementary Figure 2

Flow Cytometry sample gating strategy for CD4 T-cell populations

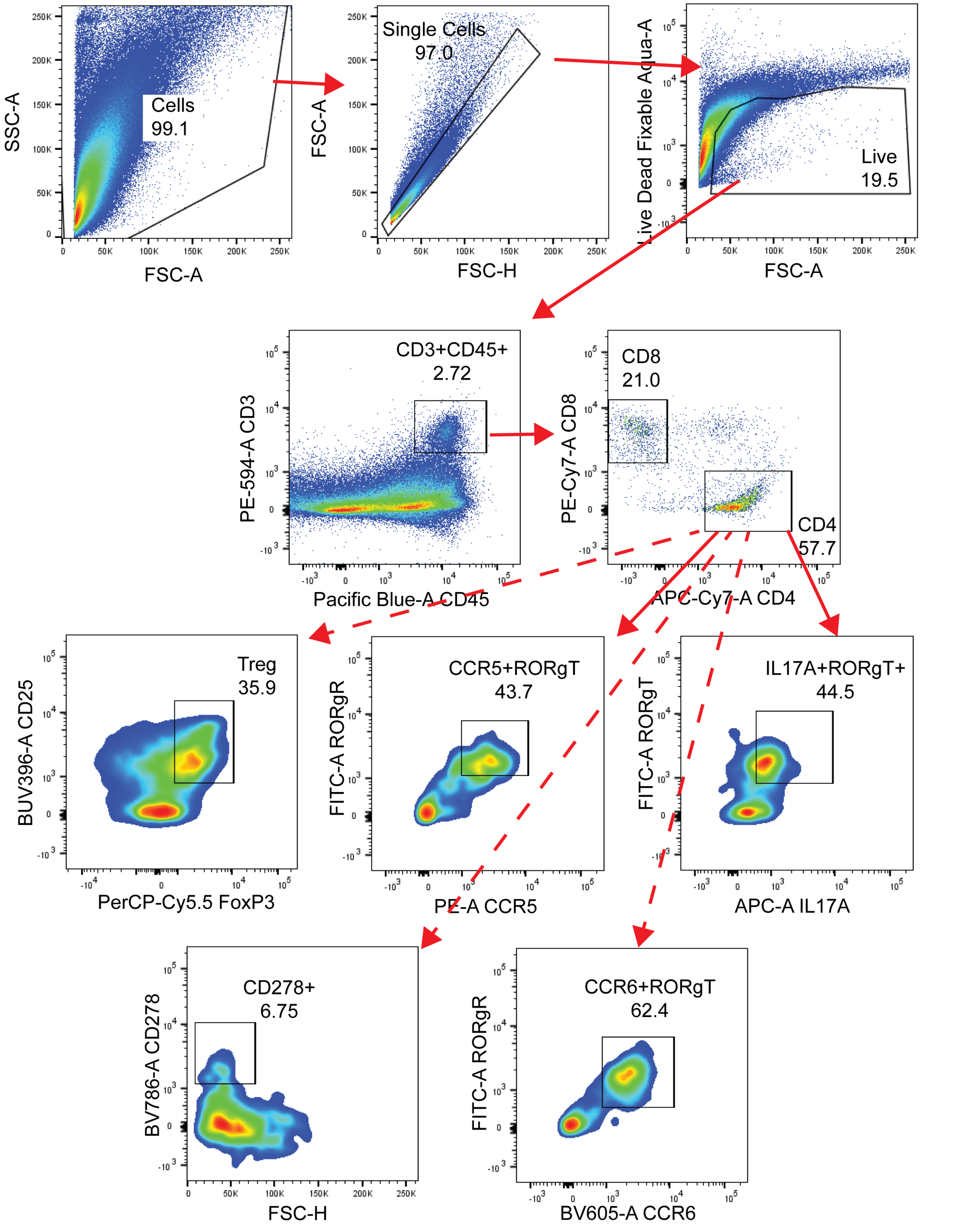

Supplementary Figure 2

Flow Cytometry sample gating strategy for APC populations

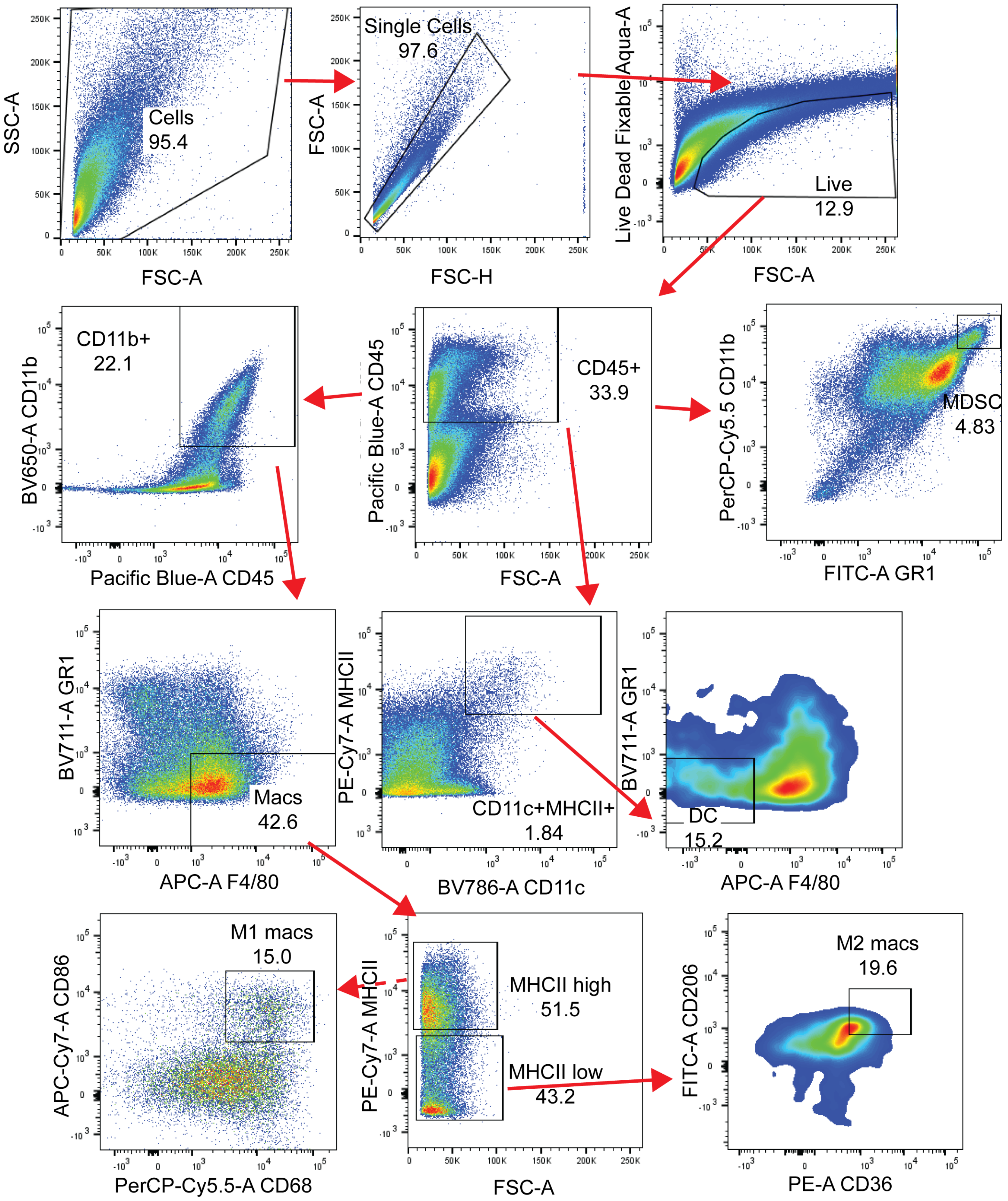

Supplementary Figure 2.

Flow cytometry sample gating strategy for indicated immune cell populations in syngeneic KP tumors including CD8+ and CD4+ T cells as well as antigen presenting cell (APC) subpopulations. Total T-cell populations gating scheme was used for Figure (2B, 2C, 2E, 2F), Figure 7A. Supplementary Figure (3A, 3B, 3E, 3G), Supplementary Figure 7A. CD4 T-cell population gating scheme was used for Figure (3C, 3D), Figure 4B, Figure 7B, Supplementary Figure 4D. APC population gating scheme used for Supplementary Figure 3F, 3H.

Supplementary Figure 3

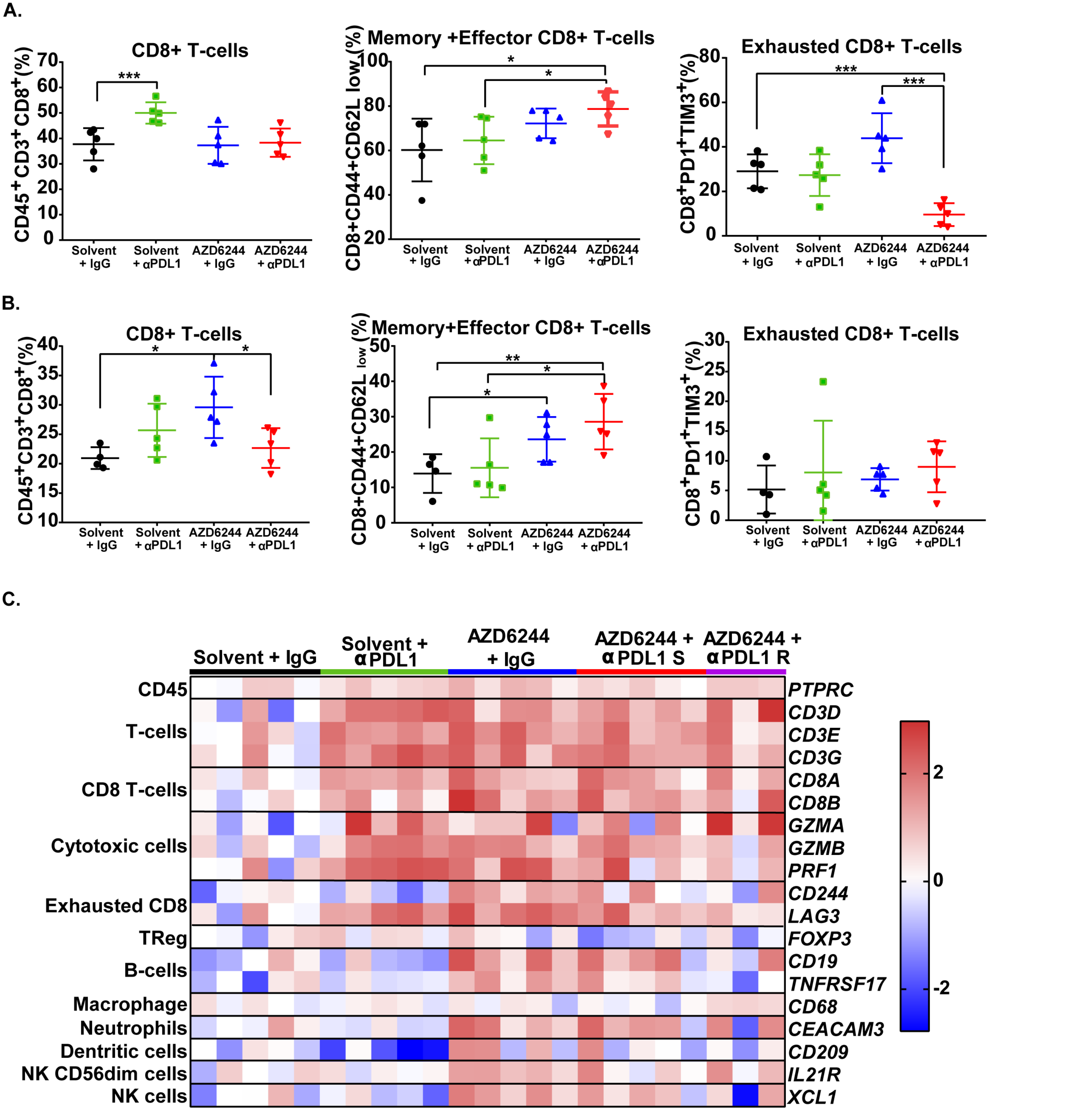

D.

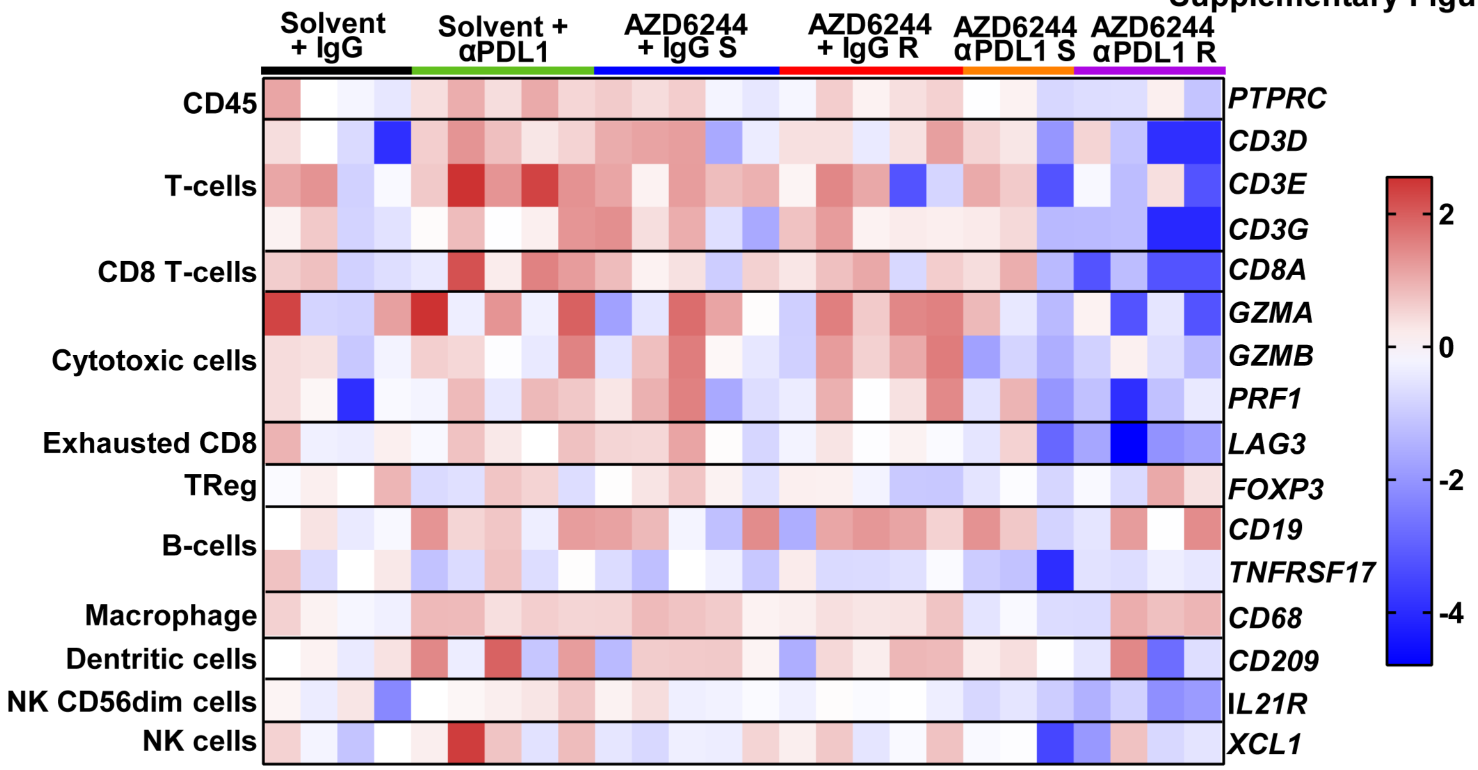
Solvent + IgG
Solvent + αPDL1
AZD6244 + IgG S
AZD6244 + IgG R
AZD6244 αPDL1 S
AZD6244 αPDL1 R
CD45
T-cells
CD8 T-cells
Cytotoxic cells
Exhausted CD8
TReg
B-cells
Macrophage
Dentritic cells
NK CD56dim cells
NK cells
PTPRC
CD3D
CD3E
CD3G
CD8A
GZMA
GZMB
PRF1
LAG3
FOXP3
CD19
TNFRSF17
CD68
CD209
IL21R
XCL1
2
0
-2
-4

E.

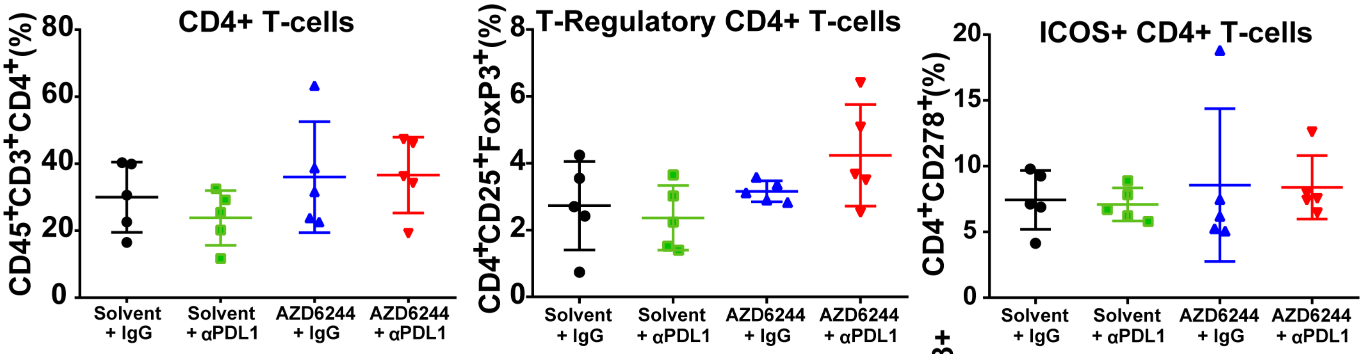
CD4+ T-cells
CD45+CD3+CD4+(%)
T-Regulatory CD4+ T-cells
CD4+CD25+FoxP3+(%)
ICOS+ CD4+ T-cells
CD4+CD278+(%)
Solvent + IgG
Solvent + αPDL1
AZD6244 + IgG
AZD6244 + αPDL1

F.

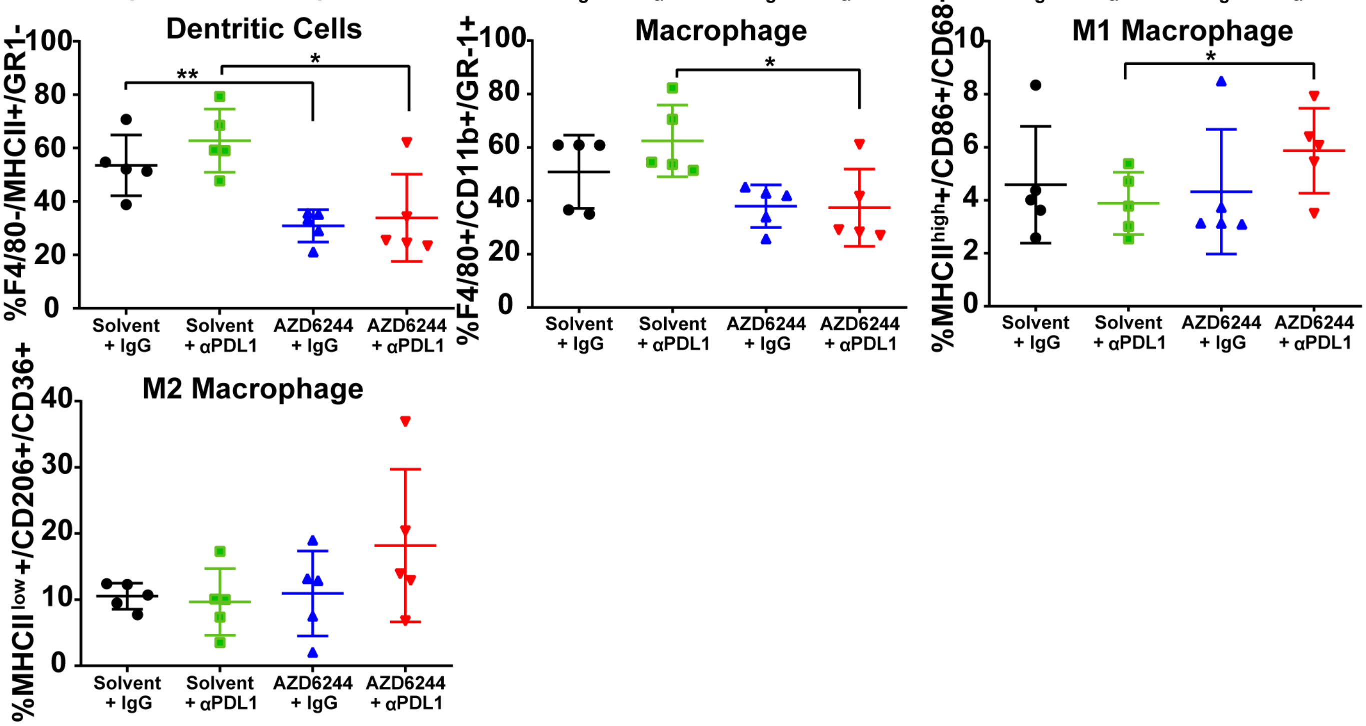
Dentritic Cells
%F4/80-/MHCII+/GR1-
Macrophage
%F4/80+/CD11b+/GR-1+
M1 Macrophage
%MHCIIhigh+/CD86+/CD68+
M2 Macrophage
%MHCIIlow+/CD206+/CD36+
**
*
Solvent + IgG
Solvent + αPDL1
AZD6244 + IgG
AZD6244 + αPDL1

Supplementary Figure 3

G.

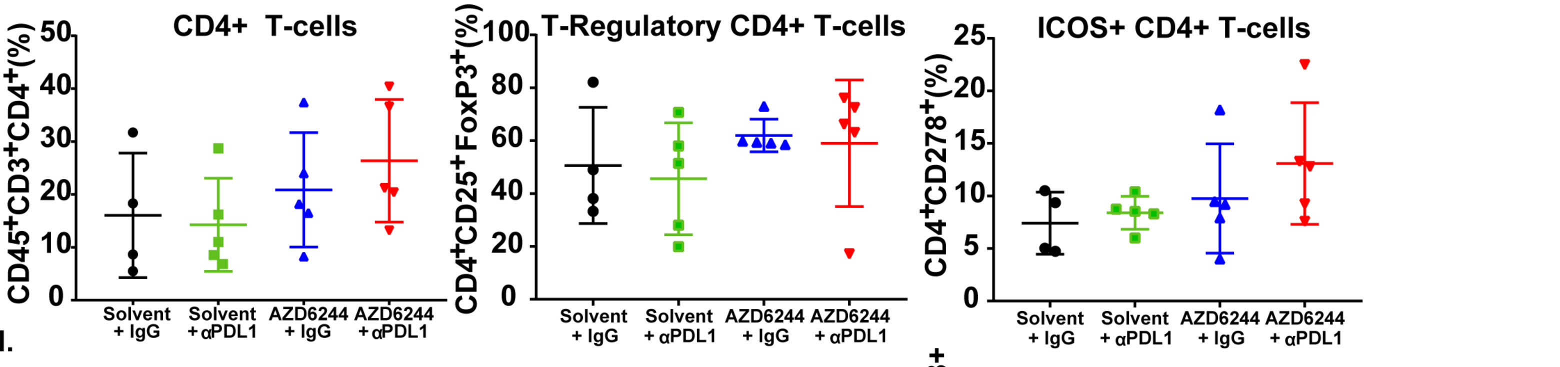

H.

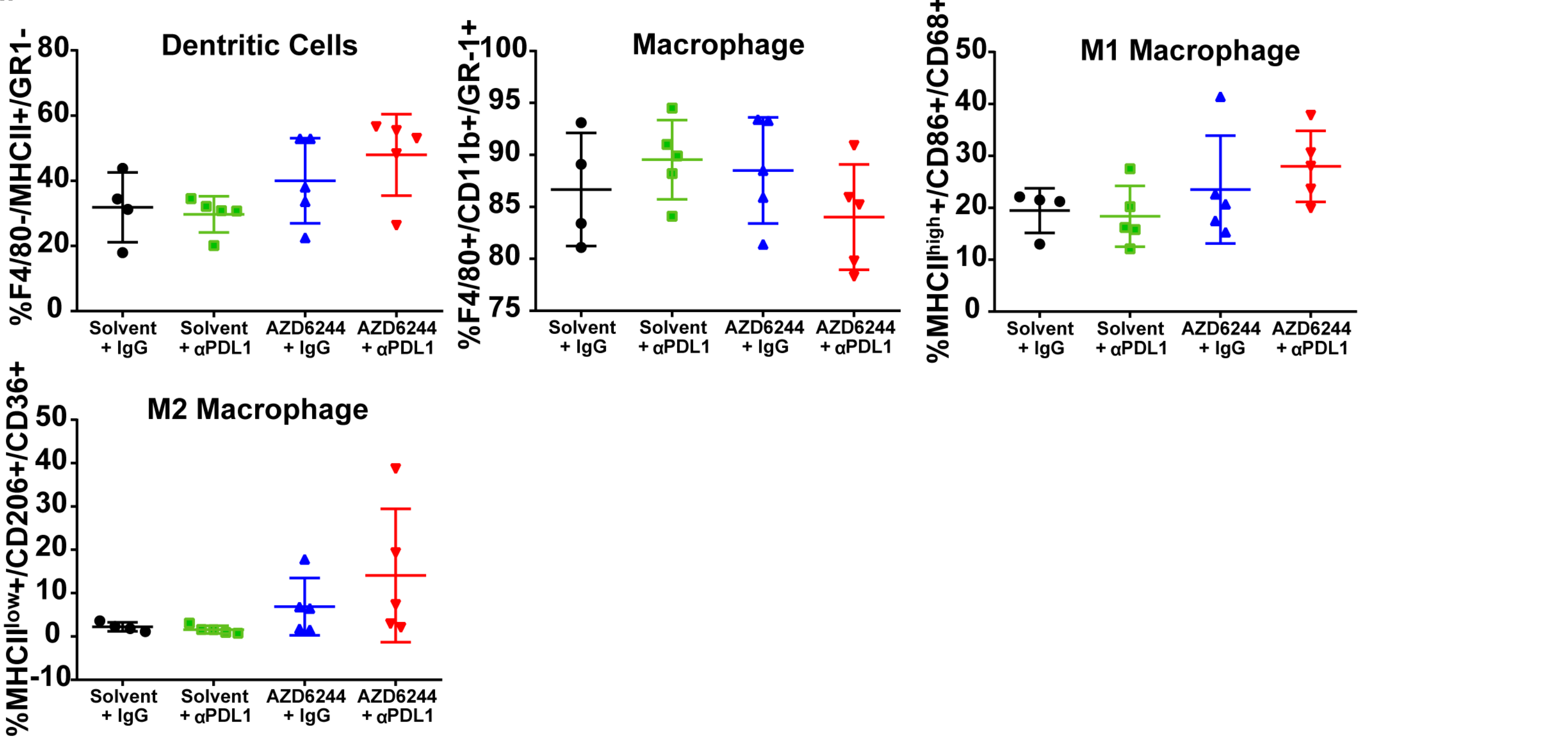

Supplementary Figure 3.

(A) Percentage of total CD8+ T-cells gated from CD45+CD3+ cells and CD8+ T-cell subpopulations gated from total CD8+ T-cells in 344SQ tumors treated with 25 mg/kg AZD6244 daily and/or weekly PD-L1 blocking antibody alone or in combination for 2 weeks. Data are presented as mean values +/-SD. n = 5. Data were analyzed using unpaired Students *t test*. *, $P < 0.05$; ***, $P < 0.001$.

(B) Percentage of total CD8+ T-cells gated from CD45+CD3+ cells and CD8+ T-cell subpopulations gated from total CD8+ T-cells using indicated markers in 393P tumors treated with 25 mg/kg AZD6244 daily and/or weekly PD-L1 blocking antibody alone or in combination for 2 weeks. Data are presented as mean values +/-SD. n = 5. Data were analyzed using unpaired Students *t test*. *, $P < 0.05$; **, $P < 0.01$.

(C) Heatmap of nanostring analysis of immune-related gene expression from 344SQ tumor tissues with indicated treatment groups at experimental endpoint from **Figure 2A**.

(D) Heatmap of nanostring analysis of immune-related gene expression from 393P tumor tissues with indicated treatment groups at experimental endpoint from **Figure 2D**.

(E) Percentage of total CD4+ T-cells gated form CD45+CD3+ cells and CD4+ T-cell subpopulations gated from total CD4+ T-cells in 344SQ tumors treated with 25 mg/kg AZD6244 daily and/or weekly PD-L1 blocking antibody alone or in combination for 2 weeks. Data are presented as mean values +/-SD. n = 5.

(F) Percentage of indicated antigen presenting cells (APC) populations gated from CD45+ cells in 344SQ tumors treated with 25 mg/kg AZD6244 daily and/or weekly PD-L1 blocking antibody alone or in combination for 2 weeks. Data are presented as mean values +/-SD. n = 5. Data were analyzed using unpaired Students *t test*. *, $P < 0.05$; **, $P < 0.01$.

(G) Percentage of total CD4+ T-cells gated form CD45+CD3+ cells and CD4+ T-cell subpopulations gated from total CD4+ T-cells in 393P tumors treated with 25 mg/kg AZD6244 daily and/or weekly PD-L1 blocking antibody alone or in combination for 2 weeks. Data are presented as mean values +/-SD. n = 5.

(H) Percentage of indicated antigen presenting cells (APC) populations gated from CD45+ cells in 393P tumors treated with 25 mg/kg AZD6244 daily and/or weekly PD-L1 blocking antibody alone or in combination for 2 weeks. Data are presented as mean values +/-SD. n = 5.

Supplementary Figure 4

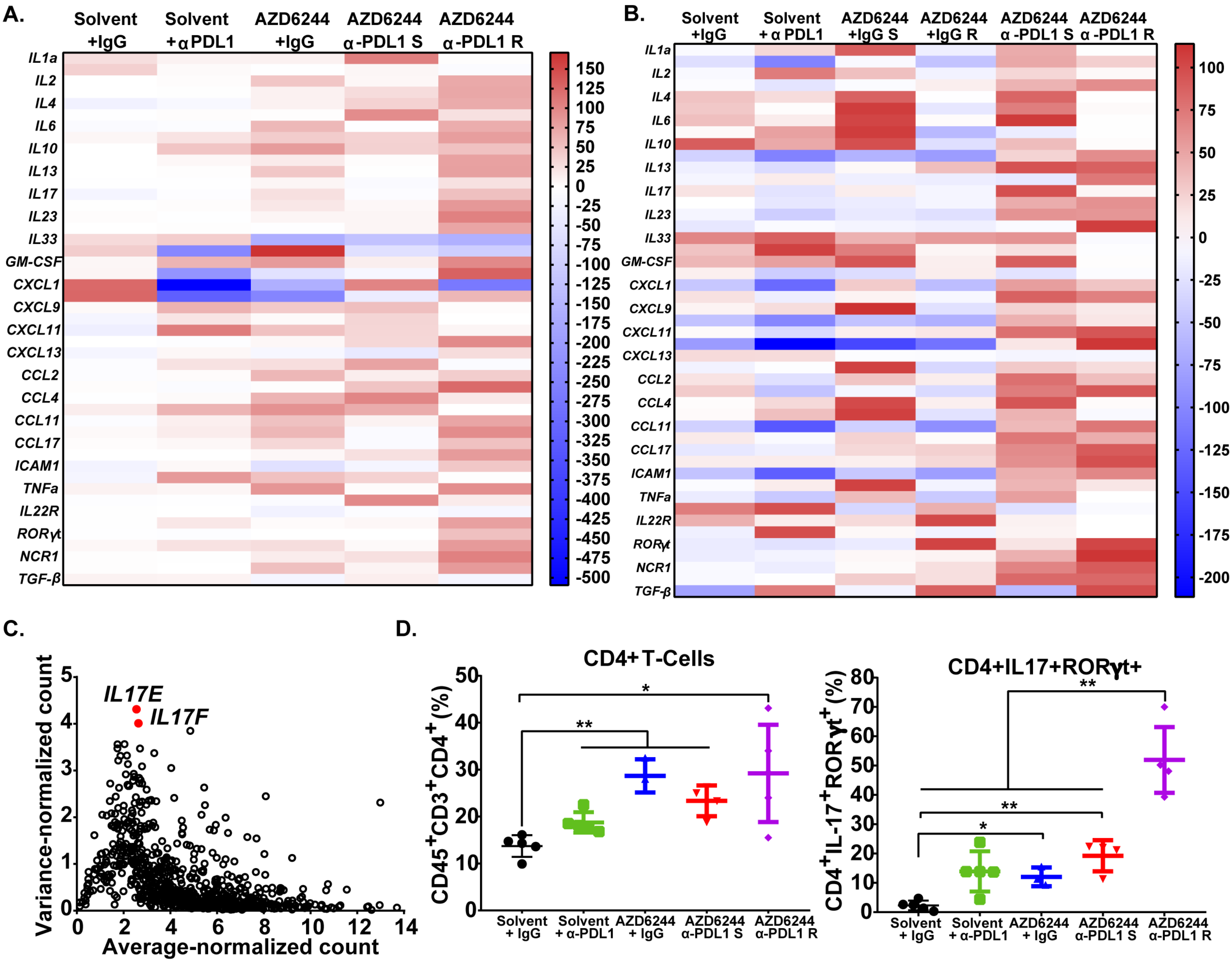

Supplementary Figure 4.

(A) Cytokine qPCR array heatmap of 344SQ tumors from the experiment in **Figure 2A**.

(B) Cytokine qPCR array heatmap of 393P tumors from the experiment in **Figure 2D**.

(C) Nanostring gene expression levels from 344SQ tumors treated with combinatorial AZD6244 and anti-PD-L1 therapies in **Figure 2A** at the point of sensitivity (Combo-S) versus point of resistance (Combo-R).

(D) Left: Percentage of total CD4+ T-cells gated from CD45+CD3+ cells in 344SQ tumors with indicated treatment from the experiment in **Figure 2A**. Right: Percentage of IL-17+RORgt+ Th17 cells gated from total CD4+ T-cells (left) in 344SQ tumors with indicated treatments from the experiment in **Figure 2A**. Data are presented as mean values +/-SD. n = 5. Data were analyzed using unpaired Students *t test*. *, $P < 0.05$; **, $P < 0.01$.

Supplementary Figure 5

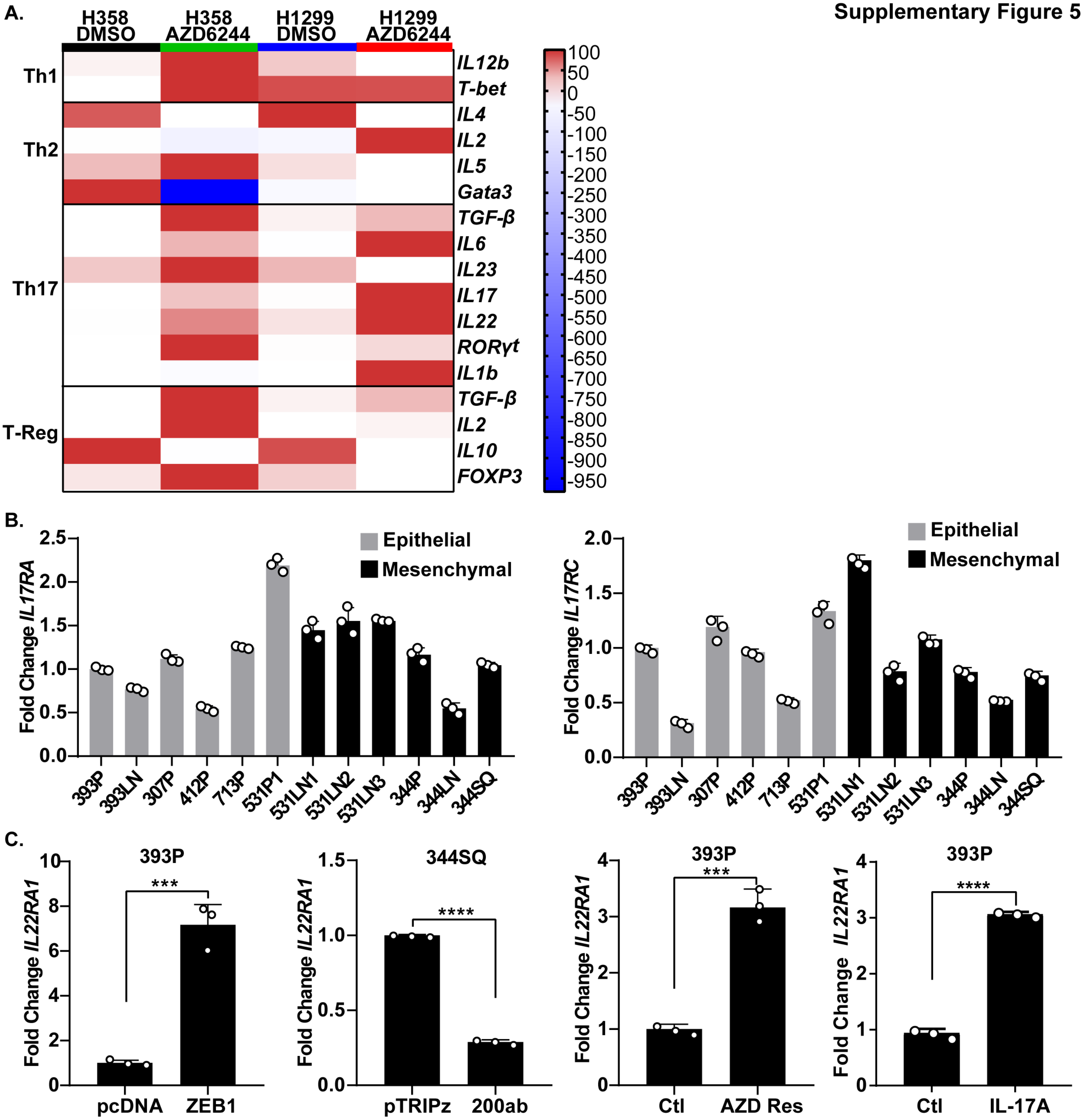

Supplementary Figure 5.

(A) Cytokine qPCR array heatmap of H358 and H1299 human lung cancer cell lines following treatment with DMSO control or 10 μM AZD6244 for 48 hrs.

(B) Left: qPCR analysis of IL17RA expression in panel of murine epithelial or mesenchymal cells. Right: qPCR analysis of IL17RC expression in panel of murine epithelial or mesenchymal cells. Data are presented as mean values +/-SD. n = 5.

(C) qPCR analysis of IL22RA1 in murine KP cell lines, left: 393P pcDNA and constituative expressing ZEB1, 344SQ pTRIPz and miR-200ab cells, 393P Ctl and AZD resistant, and 393P cells with Ctl or IL-17A. Data are presented as mean values +/-SD. n = 5. Data were analyzed using unpaired Students *t test*. ***, $P < 0.001$; ****, $P < 0.0001$.

Supplementary Figure 6

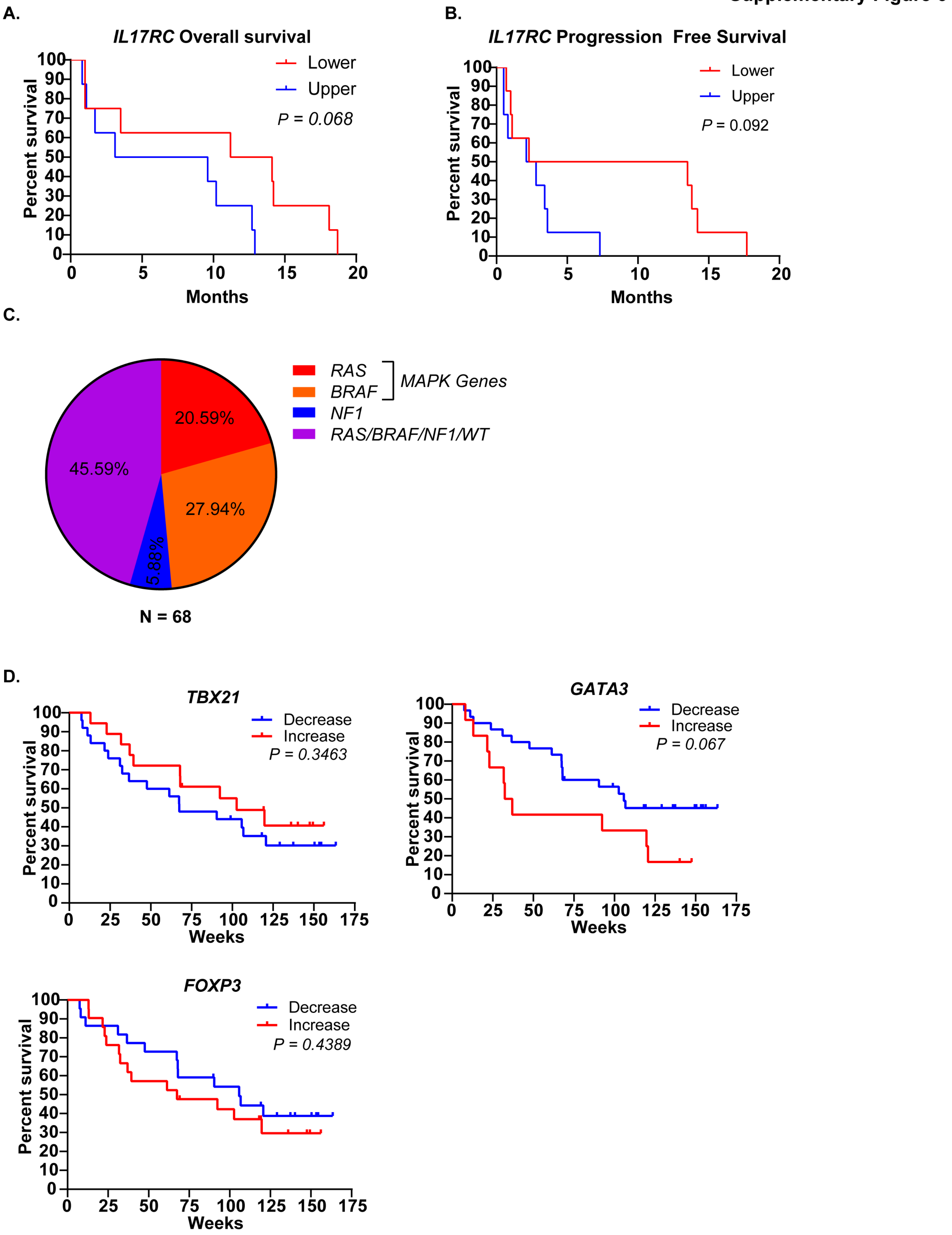

Supplementary Figure 6.

(A) Kaplan-Meier curves predicting survival of nivolumab treated non-small cell lung cancer patients based on changes of IL17RC. Statistical differences were determined using Log-rank Cox test. Dataset used GSE126044.

(B) Kaplan-Meier curves predicting progression free survival of nivolumab treated Non-small cell lung cancer patients based on changes of IL17RC. Statistical differences were determined using Log-rank Cox test. Dataset used GSE126044.

(C) Melanoma patient mutational profile from GSE91061.

(D) Kaplan-Meier curves predicting survival of nivolumab treated melanoma patients based on changes of TBX21, GATA3, and FOXP3. Statistical differences were determined using Log-rank Cox test. Dataset used GSE91061.

A.

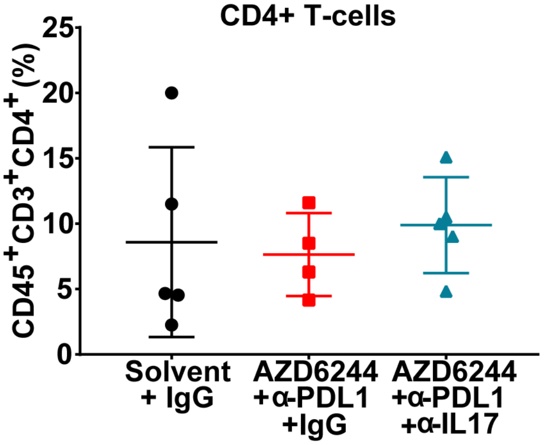
CD4+ T-cells
CD45+CD3+CD4+ (%)
25
20
15
10
5
0
Solvent + IgG
AZD6244 +α-PDL1 +IgG
AZD6244 +α-PDL1 +α-IL17

Supplementary Figure 7.

(A) Percentage of CD45+CD3+CD4+ total T-cells from tumors treated with solvent, AZD6244 + anti-PD-L1, and AZD6244+anti-PD-L1+anti-IL17. Data are presented as mean values +/-SD. n = 4-5.

Supplementary Table 1.

Antibody (clone)	Dilution used	Usage	Company/cat no
CD8 PE-Cy7 (53-6.7)	1 to 800	Flow cytometry	BioLegend/100721
CD3 PE-594 (17A2)	1 to 100	Flow cytometry	BioLegend/100246
CD62L FITC (MEL-14)	1 to 100	Flow cytometry	Tonbo/35-0621-U500
CD274 PE-594 (10F.9G2)	1 to 100	Flow cytometry	BioLegend/124323
CD44 APC (IM7)	1 to 100	Flow cytometry	BioLegend/103012
CD4 APC-Cy7 (RM4-5)	1 to 100	Flow cytometry	BioLegend/100526
FoxP3 PerCp-Cy5.5 (FJK-16s)	1 to 100	Flow cytometry	eBioscience/45-5773-82
CD86 APC-Cy7 (GL-1)	1 to 100	Flow cytometry	BioLegend/105030
CD68 PerCp-Cy5.5 (FA-11)	1 to 100	Flow cytometry	BioLegend/137009
CD69 BV650 (H1.2F3)	1 to 100	Flow cytometry	BioLegend/104541
CD45 Pacific Blue (30-F11)	1 to 100	Flow cytometry	BioLegend/103126
CD45 PerCp-Cy5.5	1 to 100	Flow cytometry	Biolegend/ 103132
CD25 BUV395 (PC61)	1 to 100	Flow cytometry	BD Biosciences/564022
CCR6 BV605 (29-2L17)	1 to 100	Flow cytometry	BioLegend/129819
CCR6 APC (29-2L17)	1 to 100	Flow cytometry	BioLegend/129814
CD11c BV786 (N418)	1 to 100	Flow cytometry	BioLegend/117335
CD44 BV711 (IM7)	1 to 100	Flow cytometry	BioLegend/103057
GR1 BV711 (RB6-8C5)	1 to 100	Flow cytometry	BioLegend/108443
CD278 PE (7E.17G9)	1 to 100	Flow cytometry	BioLegend/117406
CD278 BV785 (C398.4A)	1 to 100	Flow cytometry	BioLegend/313533
RORgt PE (AFKJS-9)	1 to 100	Flow cytometry	ThermoFisher/12-6988-82
CCR5 PE (HM-CCR5)	1 to 100	Flow cytometry	Biolegend/ 107005
TIM3 APC (B8.2c12)	1 to 100	Flow cytometry	BioLegend/134007
PD1 BV605 (29F.1A12)	1 to 100	Flow cytometry	BioLegend/135220
PD-1 FITC(29F.1A12)	1 to 100	Flow cytometry	Biolegend/ 135214
F4/80 APC (BM8.1)	1 to 100	Flow cytometry	Tonbo/20-4801-U100
MHC II/PE-Cy (M5/114.15.2)	1 to 100	Flow cytometry	BioLegend/107629
CD11b BV650 (M1170)	1 to 100	Flow cytometry	BioLegend/101239
CD206-FITC	1 to 100	Flow cytometry	Biolegend/ 141703
CD36-PE	1 to 100	Flow cytometry	Biolegend/ 102605
Zeb1	1 to 500	Western Blot	santa cruz biolabs/ sc25388
Ecad	1 to 2000	Western Blot	BD biosciences/ 610182
Ncad	1 to 500	Western Blot	BD biosciences/ 610921
PDL1	1 to 1000	Western Blot	Abcam/ ab213480
β-Actin	1 to 4,000	Western Blot	Sigma-Aldrich, A1978
Live/Dead Ghost Violet 510	1 to 100	Flow cytometry	Tonbo Biosciences/ 13-0870-T100
pCRAF	1 to 1000	Western Blot	Cell Signaling/ 9427
CRAF	1 to 1000	Western Blot	BD biosciences/ 61051
p-MEK1/2 (S217/221)	1 to 1000	Western Blot	Cell Signaling/ 9121
MEK1/2	1 to 1000	Western Blot	Cell Signaling/ CS9122S
p-ERK1/2 (T202/Y204)	1 to 1000	Western Blot	Cell Signaling/ 9101
ERK1/2	1 to 1000	Western Blot	cell signaling, CS9102S
p p90RSK	1 to 1000	Western Blot	cell signaling, 9344
pSTAT3	1 to 2000	Western Blot	cell signaling/ CS9145S
CD38	1 to 1000	Western Blot	R&D/ AF-4947
RSK	1 to 1000	Western Blot	cell signaling, 9355
RORgt (AFKJS-9)	1 to 100	IHC	Invitrogen/ 14-6988-82
Anti-mouse PDL1 (10F.9G2)	200 µg	in vivo mouse	BioXcell/BE0101
Anti-mouse IL17A	200 µg	in vivo mouse	BioXcell/BE0173
Rat IgG2b	200 µg	in vivo mouse	BioXcell/BE0086
anti-CD3 (145-2C11)	5µg/ml	co-culture	Tonbo/40-0031
anti-CD28 (37.51)	5µg/ml	co-culture	Tonbo/40-0281
HRP-conjugated mouse	1 to 3000	Western Blot	Cell Signaling/7076S
HRP-conjugated rabbit	1 to 3000	Western Blot	Cell Signaling/7074S

Supplementary Table 2.

Primer	Sequence	Species
mCCL20-F1	GTGGGTTTCACAAGACAGATGGC	murine
mCCL20-R1	CCAGTTCTGCTTTGGATCAGCG	murine
mIL6-F1	TACCACTTCACAAGTCGGAGGC	murine
mIL6-R1	CTGCAAGTGCATCATCGTTGTTC	murine
mTGFB1-F1	TGATACGCCTGAGTGGCTGTCT	murine
mTGFB1-R1	CACAAGAGCAGTGAGCGCTGAA	murine
mIL22-F1	GCTTGAGGTGTCCAACTTCCAG	murine
mIL22-R1	ACTCCTCGGAACAGTTTCTCCC	murine
mIL22RA1-F1	TTTCCTCGTCGGCTTGCTCTGT	murine
mIL22RA1-R1	CGTGTTCTTGGATGAAGCGTAGG	murine
mIL17RA-F1	CTGTATGACCTGGAGGCTTTCTG	murine
mIL17RA-R1	CGAGTAGACGATCCAGACCTTC	murine
mIL17RC-F1	TAGAGCCAGACTCTGAGAGGGT	murine
mIL17RC-R1	AAGGCGCATCTAGCTGCCATAC	murine
mIL17A-F1	CAGACTACCTCAACCGTTCCAC	murine
mIL17A-R1	TCCAGCTTTCCCTCCGCATTGA	murine
mIL23a-F1	CATGCTAGCCTGGAACGCACAT	murine
mIL23a-R1	ACTGGCTGTTGTCCTTGAGTCC	murine
mIL23R-F1	GTCCACCAAACTTCCCAGACAG	murine
mIL23R-R1	CCTGAAGCAGGATGTCCTCTGA	murine
mIL33-F1	CTACTGCATGAGACTCCGTTCTG	murine
mIL33-R1	AGAATCCCGTGGATAGGCAGAG	murine
mIL21-F1	GCCTCCTGATTAGACTTCGTCAC	murine
mIL21-R1	CAGGCAAAAGCTGCATGCTCAC	murine
mTbx21-F1	CCACCTGTTGTGGTCCAAGTTC	murine
mTbx21-R1	CCACAAACATCCTGTAATGGCTTG	murine
mIL25-F1	TGGCTGAAGTGGAGCTCTGCAT	murine
mIL25-R1	CCCGATTCAAGTCCCTGTCCAA	murine
mGata3-F1	CCTCTGGAGGAGGAACGCTAAT	murine
mGata3-R1	GTTTCGGGTCTGGATGCCTTCT	murine
mBcl6-F1	CAGAGATGTGCCTCCATACTGC	murine
mBcl6-R1	CTCCTCAGAGAAACGGCAGTCA	murine
mCD19-F1	GCCACAGCTTTAGATGAAGGCAC	murine
mCD19-R1	CATCCACCAGTTCTCAACAGCC	murine
mCXCL13-F1	CATAGATCGGATTCAAGTTACGCC	murine
mCXCL13-R1	GTAACCATTTGGCACGAGGATTC	murine
mG-CSF-F1	ATCCCGAAGGCTTCCCTGAGTG	murine
mG-CSF-R1	AGGAGACCTTGGTAGAGGCAGA	murine
mGM-CSF-F1	AACCTCCTGGATGACATGCCTG	murine
mGM-CSF-R1	AAATTGCCCCGTAGACCCTGCT	murine
mCCL1-F1	GCTTACGGTCTCCAATAGCTGC	murine
mCCL1-R1	GCTTTCTCTACCTTTGTTCAGCC	murine
mCCL11-F1	TCCATCCCAACTTCCTGCTGCT	murine
mCCL11-R1	CTCTTTGCCCAACCTGGTCTTG	murine
mICAM1-F1	AAACCAGACCCTGGAACTGCAC	murine
mICAM1-R1	GCCTGGCATTTCAGAGTCTGCT	murine

mIFNg-F1	CAGCAACAGCAAGGCGAAAAAGG	murine
mIFNg-R1	TTTCCGCTTCCTGAGGCTGGAT	murine
mIL1a-F1	ACGGCTGAGTTTCAGTGAGACC	murine
mIL1a-R1	CACTCTGGTAGGTGTAAGGTGC	murine
mIL1b-F1	TGGACCTTCCAGGATGAGGACA	murine
mIL1b-R1	GTTCATCTCGGAGCCTGTAGTG	murine
mIL2-F1	GCGGCATGTTCTGGATTTGACTC	murine
mIL2-R1	CCACCACAGTTGCTGACTCATC	murine
mIL3-F1	CCTGCCTACATCTGCGAATGAC	murine
mIL3-R1	GAGGTTAGCACTGTCTCCAGATC	murine
mIL4-F1	ATCATCGGCATTTTGAACGAGGTC	murine
mIL4-R1	ACCTTGGAAGCCCTACAGACGA	murine
mIL5-F1	GATGAGGCTTCCTGTCCCTACT	murine
mIL5-R1	TGACAGGTTTTGGAATAGCATTTCC	murine
mIL7-F1	CAGGAACTGATAGTAATTGCCCG	murine
mIL7-R1	CTTCAACTTGCGAGCAGCACGA	murine
mIL10-F1	CGGGAAGACAATAACTGCACCC	murine
mIL10-R1	CGGTTAGCAGTATGTTGTCCAGC	murine
mIL12B-F1	TTGAACTGGCGTTGGAAGCACG	murine
mIL12B-R1	CCACCTGTGAGTTCTTCAAAGGC	murine
mIL16-F1	CACGCAGACTTCATCCTCCACA	murine
mIL16-R1	AGCTATAGTCCATCCGTGCCTG	murine
mIL17F-F1	AACCAGGGCATTTCTGTCCCAC	murine
mIL17F-R1	GGCATTGATGCAGCCTGAGTGT	murine
mIL27-F1	TCTCGATTGCCAGGAGTGAACC	murine
mIL27-R1	AGTGTGGTAGCGAGGAAGCAGA	murine
mCXCL1-F1	TCCAGAGCTTGAAGGTGTTGCC	murine
mCXCL1-R1	AACCAAGGGAGCTTCAGGGTCA	murine
mM-CSF-F1	GCCTCCTGTTCTACAAGTGGAAG	murine
mM-CSF-R1	ACTGGCAGTTCCACCTGTCTGT	murine
mCCL12-F1	GCTACAGGAGAATCACAAGCAGC	murine
mCCL12-R1	ACGTCTTATCCAAGTGGTTTATGG	murine
mCCL3-F1	ACTGCCTGCTGCTTCTCCTACA	murine
mCCL3-R1	ATGACACCTGGCTGGGAGCAAA	murine
mCCL4-F1	ACCCTCCCACTTCCTGCTGTTT	murine
mCCL4-R1	CTGTCTGCCTCTTTTGGTCAGG	murine
mCXCL12-F1	CATCCAGAGCTTGAGTGTGACG	murine
mCXCL12-R1	GGCTTCAGGGTCAAGGCAAACT	murine
mCCL5-F1	CCTGCTGCTTTGCCTACCTCTC	murine
mCCL5-R1	ACACACTTGGCGGTTCCTTCGA	murine
mCXCL12-F1	GGAGGATAGATGTGCTCTGGAAC	murine
mCXCL12-R1	AGTGAGGATGGAGACCGTGGTG	murine
mCCL17-F1	CGAGAGTGCTGCCTGGATTACT	murine
mCCL17-R1	GGTCTGCACAGATGAGCTTGCC	murine
mTNFa-F1	GGTGCCTATGTCTCAGCCTCTT	murine
mTNFa-R1	GCCATAGAACTGATGAGAGGGAG	murine
mPDL1-F1	TGCGGACTACAAGCGAATCACG	murine

mPDL1-R1	CTCAGCTTCTGGATAACCCTCG	murine
mIL13-F1	AACGGCAGCATGGTATGGAGTG	murine
mIL13-R1	TGGGTCCTGTAGATGGCATTGC	murine
mRORc-F1	GTGGAGTTTGCCAAGCGGCTTT	murine
mRORc-R1	CCTGCACATTCTGACTAGGACG	murine
mFOXP3-F1	CCTGGTTGTGAGAAGGTCTTCG	murine
mFOXP3-R1	TGCTCCAGAGACTGCACCACTT	murine
mNCR1-F1	TAGGGCTCACAGAGGGACATAC	murine
mNCR1-R1	GTAGGTGCAAGGCTGCTGTTCT	murine
mCXCL9-F1	CCTAGTGATAAGGAATGCACGATG	murine
mCXCL9-R1	CTAGGCAGGTTTGATCTCCGTTC	murine
mCXCL11-F1	CCGAGTAACGGCTGCGACAAAG	murine
mCXCL11-R1	CCTGCATTATGAGGCGAGCTTG	murine
mCXCL13-F1	CATAGATCGGATTCAAGTTACGCC	murine
mCXCL13-R1	GTAACCATTTGGCACGAGGATTC	murine
mCCL2-F1	GCTACAAGAGGATCACCAGCAG	murine
mCCL2-R1	GTCTGGACCCATTCCTTCTTGG	murine
hIFNG F	GAGTGTGGAGACCATCAAGGAAG	human
hIFNG R	TGCTTTGCGTTGGACATTCAAGTC	human
hIL12B F	GACATTCTGCGTTCAGGTCCAG	human
hIL12B R	CATTTTTGCGGCAGATGACCGTG	human
hIL12RB2 F	AGACCTCAGTGGTGTAGCAGAG	human
hIL12RB2 R	TGATGACCAGCGGTTCAGGATC	human
hTBX21 F	ATTGCCGTGACTGCCTACCAGA	human
hTBX21 R	GGAATTGACAGTTGGGTCCAGG	human
hIL4 F	CCGTAACAGACATCTTTGCTGCC	human
hIL4 R	GAGTGTCCTTCTCATGGTGGCT	human
hIL33 F	GCCTGTCAACAGCAGTCTACTG	human
hIL33 R	TGTGCTTAGAGAAGCAAGATACTC	human
hIL5 F	GGAATAGGCACACTGGAGAGTC	human
hIL5 R	CTCTCCGTCTTTCTTCTCCACAC	human
hGATA3 F	ACCACAACCACACTCTGGAGGA	human
hGATA3 R	TCGGTTTCTGGTCTGGATGCCT	human
hIL13 F	ACGGTCATTGCTCTCACTTGCC	human
hIL13 R	CTGTCAGGTTGATGCTCCATACC	human
hIL2 F	AGAACTCAAACCTCTGGAGGAAG	human
hIL2 R	GCTGTCTCATCAGCATATTCACAC	human
hIL6 F	AGACAGCCACTCACCTCTTCAG	human
hIL6 R	TTCTGCCAGTGCCTCTTTGCTG	human
hIL23R F	AGGTACTGGCAGCCTTGGAGTT	human
hIL23R R	CCCTGTAGAGATGGAAGCAACTG	human
hIL17A F	CGGACTGTGATGGTCAACCTGA	human
hIL17A R	GCACTTTGCCTCCCAGATCACA	human
hIL22 F	GTTCCAGCCTTATATGCAGGAGG	human
hIL22 R	GCACATTCCTCTGGATATGCAGG	human
hRORC F	GAGGAAGTGACTGGCTACCAGA	human
hRORC R	GCACAATCTGGTCATTCTGGCAG	human

hIL17F F	AACCAGCGCGTTTCCATGTCAC	human
hIL17F R	GAGCATTGATGCAGCCCAAGTTC	human
hIL21 F	CCAAGGTCAAGATCGCCACATG	human
hIL21 R	TGGAGCTGGCAGAAATTCAGGG	human
hIL1b F	CCACAGACCTTCCAGGAGAATG	human
hIL1b R	GTGCAGTTCAGTGATCGTACAGG	human
hTGFb1 F	TACCTGAACCCGTGTTGCTCTC	human
hTGFb1 R	GTTGCTGAGGTATCGCCAGGAA	human
hFOXP3 F	GGCACAATGTCTCCTCCAGAGA	human
hFOXP3 R	CAGATGAAGCCTTGGTCAGTGC	human
hIL10 F	TCTCCGAGATGCCTTCAGCAGA	human
hIL10 F	TCAGACAAGGCTTGGCAACCCA	human
mGata3 F	CCTCTGGAGGAGGAACGCTAAT	human
mGata3 R	GTTTCGGGTCTGGATGCCTTCT	human
mCcr6 F	ACAGAGCCATCCGAGTCGTGAT	human
mCcr6 R	CTGGTGTAGGCGAGGACTTTCT	human
mCcr5 F	GTCTACTTTCTCTTCTGGACTCC	human
mCcr5 R	CCAAGAGTCTCTGTTGCCTGCA	human
