# Supplementary material for: Th17 cells contribute to combination MEK inhibitor and anti-PD-L1 therapy resistance in KRAS/p53 mutant lung cancers
Source: Nat Commun. 2021 May 10;12:2606. doi: 10.1038/s41467-021-22875-w (PMC8110980; doi:10.1038/s41467-021-22875-w)
Supplement: Supplementary file 3 — Reporting Summary [file 41467_2021_22875_MOESM3_ESM.pdf]

nature research

Corresponding author(s): Don L. Gibbons, M.D., Ph.D.

Last updated by author(s): Mar 12, 2021

Reporting Summary

Nature Research wishes to improve the reproducibility of the work that we publish. This form provides structure for consistency and transparency in reporting. For further information on Nature Research policies, see our Editorial Policies and the Editorial Policy Checklist.

Statistics

For all statistical analyses, confirm that the following items are present in the figure legend, table legend, main text, or Methods section.

n/a	Confirmed	
[ ]	[x]	The exact sample size (*n*) for each experimental group/condition, given as a discrete number and unit of measurement
[ ]	[x]	A statement on whether measurements were taken from distinct samples or whether the same sample was measured repeatedly
[ ]	[x]	The statistical test(s) used AND whether they are one- or two-sided<br>*Only common tests should be described solely by name; describe more complex techniques in the Methods section.*
[x]	[ ]	A description of all covariates tested
[ ]	[x]	A description of any assumptions or corrections, such as tests of normality and adjustment for multiple comparisons
[ ]	[x]	A full description of the statistical parameters including central tendency (e.g. means) or other basic estimates (e.g. regression coefficient) AND variation (e.g. standard deviation) or associated estimates of uncertainty (e.g. confidence intervals)
[ ]	[x]	For null hypothesis testing, the test statistic (e.g. *F*, *t*, *r*) with confidence intervals, effect sizes, degrees of freedom and *P* value noted<br>*Give P values as exact values whenever suitable.*
[x]	[ ]	For Bayesian analysis, information on the choice of priors and Markov chain Monte Carlo settings
[x]	[ ]	For hierarchical and complex designs, identification of the appropriate level for tests and full reporting of outcomes
[ ]	[x]	Estimates of effect sizes (e.g. Cohen's *d*, Pearson's *r*), indicating how they were calculated

Our web collection on statistics for biologists contains articles on many of the points above.

Software and code

Policy information about availability of computer code

Data collection	GraphPad 8 was used to input data and generate graphs, FACSDiva v8.0.1was used to collect FACS data, Aperio AT2 Scanner and ImageScope software version 12.3.3 was used to scan and capture histology images, BioTek Epoch Microplate Spectrophotometer was used to measure colorimetric cell growth assays, R 3.5.1 was used to analyze RPPA, RNA profiling, and TCGA data. Westerns were developed by radiographic film. QPCR data were collected using Applied Biosystems 7500 Fast.
Data analysis	GraphPad 8 was used to analyze fold changes and statistical data on graphs. R v3.4.2 & v3.5.1was used to analyze RPPA, RNA profiling, and TCGA datasets. FlowJo v.10.5.3 & v.10.6.1 was used to analyze FACS data.

For manuscripts utilizing custom algorithms or software that are central to the research but not yet described in published literature, software must be made available to editors and reviewers. We strongly encourage code deposition in a community repository (e.g. GitHub). See the Nature Research guidelines for submitting code & software for further information.

Data

Policy information about availability of data

All manuscripts must include a data availability statement. This statement should provide the following information, where applicable:

- Accession codes, unique identifiers, or web links for publicly available datasets
- A list of figures that have associated raw data
- A description of any restrictions on data availability

The RNA sequence data that support the findings of this study are available in GEO-NCBI with the accession codes GSE126044 and GSE91061. The authors declare that all other data supporting findings in this study are available within the paper. http://DOI: 10.1038/s12276-020-00493-8. https://www.ncbi.nlm.nih.gov/geo/query/acc.cgi.

Field-specific reporting

Please select the one below that is the best fit for your research. If you are not sure, read the appropriate sections before making your selection.

- [x] Life sciences
- [ ] Behavioural & social sciences
- [ ] Ecological, evolutionary & environmental sciences

For a reference copy of the document with all sections, see nature.com/documents/nr-reporting-summary-flat.pdf

Life sciences study design

All studies must disclose on these points even when the disclosure is negative.

Sample size	Sample size for mice experiments was determined from previous studies with the Kras;p53 models (Peng et. al. STM PMID:30867319) (Chen et. al. Cancer Discov PMID:30012853) which generated sufficient statistical power within acceptable variation where N = 4 to 5 mice per group. In vitro studies sample sizes were also determined from prior studies (Chen et. al. Nat Commun PMID:25348003) (Peng et al. Nat Commun PMID:32908154) with similar models where N = 3 to 8 technical replicates.
Data exclusions	Animals that did not develop tumors after implantation or induction or that died for reasons not related to tumor burden were not included in data analyses.
Replication	All attempts at replication were successful. In vitro experiments were replicated at least three independent times. In vivo studies were replicated twice in order to collect sufficient tissues for all necessary downstream experiments.
Randomization	When tumors reached appropriate size as reported in the Methods Section, mice were randomized into indicated treatment groups. For all other experiments, samples were allocated into experimental groups by randomization.
Blinding	Mice treatments were not blinded since most of the experiments required daily treatments and treatment groups and mice cage numbers had to be known for investigators. Blinding was not relevant to the remaining experiments described in the study due to primary investigators performing experiments from start to finish due to technicality required.

Reporting for specific materials, systems and methods

We require information from authors about some types of materials, experimental systems and methods used in many studies. Here, indicate whether each material, system or method listed is relevant to your study. If you are not sure if a list item applies to your research, read the appropriate section before selecting a response.

Materials & experimental systems

n/a	Involved in the study	
[ ]	[x]	Antibodies
[ ]	[x]	Eukaryotic cell lines
[x]	[ ]	Palaeontology and archaeology
[ ]	[x]	Animals and other organisms
[x]	[ ]	Human research participants
[x]	[ ]	Clinical data
[x]	[ ]	Dual use research of concern

Methods

n/a	Involved in the study	
[x]	[ ]	ChIP-seq
[ ]	[x]	Flow cytometry
[x]	[ ]	MRI-based neuroimaging

Antibodies

Antibodies used

Antibody (clone) Dilution used Usage Company/cat no
CD8 PE-Cy7 (53-6.7) 1 to 800 Flow cytometry BioLegend/100721
CD3 PE-594 (17A2) 1 to 100 Flow cytometry BioLegend/100246
CD62L FITC (MEL-14) 1 to 100 Flow cytometry Tonbo/35-0621-U500
CD274 PE-594 (10F.9G2) 1 to 100 Flow cytometry BioLegend/124323
CD44 APC (IM7) 1 to 100 Flow cytometry BioLegend/103012
CD4 APC-Cy7 (RM4-5) 1 to 100 Flow cytometry BioLegend/100526
FoxP3 PerCp-Cy5.5 (FJK-16s) 1 to 100 Flow cytometry eBioscience/45-5773-82
CD86 APC-Cy7 (GL-1) 1 to 100 Flow cytometry BioLegend/105030
CD68 PerCp-Cy5.5 (FA-11) 1 to 100 Flow cytometry BioLegend/137009
CD69 BV650 (H1.2F3) 1 to 100 Flow cytometry BioLegend/104541
CD45 Pacific Blue (30-F11) 1 to 100 Flow cytometry BioLegend/103126
CD45 PerCp-Cy5.5 1 to 100 Flow cytometry Biolegend/ 103132
CD25 BUV395 (PC61) 1 to 100 Flow cytometry BD Biosciences/564022

CCR6 BV605 (29-2L17) 1 to 100 Flow cytometry BioLegend/129819
CCR6 APC (29-2L17) 1 to 100 Flow cytometry BioLegend/129814
CD11c BV786 (N418) 1 to 100 Flow cytometry BioLegend/117335
CD44 BV711 (IM7) 1 to 100 Flow cytometry BioLegend/103057
GR1 BV711 (RB6-8C5) 1 to 100 Flow cytometry BioLegend/108443
CD278 PE (7E.17G9) 1 to 100 Flow cytometry BioLegend/117406
CD278 BV785 (C398.4A) 1 to 100 Flow cytometry BioLegend/313533
RORgt PE (AFKJS-9) 1 to 100 Flow cytometry ThermoFisher/12-6988-82
CCR5 PE (HM-CCR5) 1 to 100 Flow cytometry Biolegend/ 107005
TIM3 APC (B8.2c12) 1 to 100 Flow cytometry BioLegend/134007
PD1 BV605 (29F.1A12) 1 to 100 Flow cytometry BioLegend/135220
PD-1 FITC(29F.1A12) 1 to 100 Flow cytometry Biolegend/ 135214
F4/80 APC (BM8.1) 1 to 100 Flow cytometry Tonbo/20-4801-U100
MHC II/PE-Cy (M5/114.15.2) 1 to 100 Flow cytometry BioLegend/107629
CD11b BV650 (M1170) 1 to 100 Flow cytometry BioLegend/101239
CD206-FITC 1 to 100 Flow cytometry Biolegend/ 141703
CD36-PE 1 to 100 Flow cytometry Biolegend/ 102605
Zeb1 1 to 500 Western Blot santa cruz biolabs/ sc25388
Ecad 1 to 5000 Western Blot BD biosciences/ 610182
Ncad 1 to 500 Western Blot BD biosciences/ 610921
PDL1 1 to 1000 Western Blot Abcam/ ab213480
β-Actin 1 to 4,000 Western Blot Sigma-Aldrich, A1978
Live/Dead Ghost Violet 510 1 to 100 Flow cytometry Tonbo Biosciences/ 13-0870-T100
pCRAF 1 to 1000 Western Blot Cell Signaling/ 9427
CRAF 1 to 1000 Western Blot BD biosciences/ 61051
p-MEK1/2 (S217/221) 1 to 1000 Western Blot Cell Signaling/ 9121
MEK1/2 1 to 1000 Western Blot Cell Signaling/ CS9122S
p-ERK1/2 (T202/204) 1 to 1000 Western Blot Cell Signaling/ 9101
ERK1/2 1 to 1000 Western Blot cell signaling, CS9102S
p p90RSK 1 to 1000 Western Blot cell signaling, 9344
pSTAT3 1 to 2000 Western Blot cell signaling/ CS9145S
CD38 1 to 1000 Western Blot R&D/ AF-4947
RSK 1 to 1000 Western Blot cell signaling, 9355
RORgt (AFKJS-9) 1 to 100 IHC Invitrogen/ 14-6988-82
Anti-mouse PDL1 (10F.9G2) 200 µg in vivo mouse BioXcell/BE0101
Anti-mouse IL17A 200 µg in vivo mouse BioXcell/BE0173
Rat IgG2b 200 µg in vivo mouse BioXcell/BE0086
anti-CD3 (145-2C11) 5µg/ml co-culture Tonbo/40-0031
anti-CD28 (37.51) 5µg/ml co-culture Tonbo/40-0281
HRP-conjugated mouse 1 to 3000 Western Blot Cell Signaling/7076S
HRP-conjugated rabbit 1 to 3000 Western Blot Cell Signaling/7074S

Validation

Antibodies have been validated either from prior reports and studies or validated by the manufacturer as stated on the website from the catalog numbers listed above or published references on the manufacturers' websites. Manufacturer states the antibody has been validated for intended use. Manufacturer citation are listed in manufacturer website for each specific antibody.

Eukaryotic cell lines

Policy information about cell lines

Cell line source(s)

Murine KP cell lines (344SQ, 393P, 393LN, 307P, 412P, 713P, 531P1, 531LN1, 531LN2, 531LN3, 344P, 344LN) were by culturing tumor nodules onto cell culture dish per Gibbons et al 2009. DOI: 10.1101/gad.1820209. Human cell lines H2122, H1299, H358, H157, and A549 were obtained from ATCC.

Authentication

Cell lines were authenticated in previous publications when the cells were initially derived. No new authentications were performed in this study.

Mycoplasma contamination

All cell lines were tested to be mycoplasma negative and were tested monthly using commercial detection kits as described in the Methods section.

Commonly misidentified lines
(See ICLAC register)

No misidentified cell lines were used.

Animals and other organisms

Policy information about studies involving animals; ARRIVE guidelines recommended for reporting animal research

Laboratory animals

Mice were 129/sv both male and female between the ages of 6 to 8 weeks at the start of each experiment. Mice were housed in ventilated cage enclosures in an environment maintained at 50% humidity with ambient temperatures range between 66 °F and 78 °

	F and 12-h-light/dark cycles.
Wild animals	Study did not involve wild animals
Field-collected samples	Study did not utilize field-collected samples.
Ethics oversight	All animal experiments were approved by the Institutional Animal Care and Use Committee at The University of Texas MD Anderson Cancer Center.

Note that full information on the approval of the study protocol must also be provided in the manuscript.

Flow Cytometry

Plots

Confirm that:

- [x] The axis labels state the marker and fluorochrome used (e.g. CD4-FITC).
- [x] The axis scales are clearly visible. Include numbers along axes only for bottom left plot of group (a 'group' is an analysis of identical markers).
- [x] All plots are contour plots with outliers or pseudocolor plots.
- [x] A numerical value for number of cells or percentage (with statistics) is provided.

Methodology

Sample preparation	Cell samples were collected from primary murine subcutaneous tumor tissues, digested with collagenase/dispase solution, and passed through single-cell filters
Instrument	FACS data was collected using BD LSRFortessa flow cytometer.
Software	FACS data was collected using FACSDiva and analyzed using FlowJo.
Cell population abundance	FACS analysis was performed on each sample to a total cell number between 500,000 to 2,000,000 events with a threshold of 10,000 to increase quality of samples per event. Each gated population was sorted so that at least 1,000 cells for the furthest gated cell population was recorded to obtain satisfactory percentage of the cell population. FACS quality was also ensured using compensation controls and FMO controls to verify that observed and gated populations were accurate and distinct.
Gating strategy	All samples were gated for FSC/SSC, then single cell gated for FSC-A/FSC-H, then gated for CD45+ cells. For T-cell populations, CD45+ were double-gated for CD3+ cells. CD45+CD3+ cells were then gated for CD4+ or CD8+ cells from the same populations. CD8+ cells were gated for the indicated populations listed in the graphs from the figures. For antigen presenting populations, CD45+ cells were gated for the populations indicated on the graphs in the figures. Gates were drawn from distinct, observable stained populations using prior FMO controls to indicate where to draw the positive gates that were separate from the negative populations.

- [x] Tick this box to confirm that a figure exemplifying the gating strategy is provided in the Supplementary Information.
